# SNOWED: Automatically Constructed Dataset of Satellite Imagery for Water Edge Measurements

**DOI:** 10.3390/s23094491

**Published:** 2023-05-05

**Authors:** Gregorio Andria, Marco Scarpetta, Maurizio Spadavecchia, Paolo Affuso, Nicola Giaquinto

**Affiliations:** Department of Electrical and Information Engineering, Polytechnic University of Bari, Via E. Orabona 4, 70125 Bari, Italynicola.giaquinto@poliba.it (N.G.)

**Keywords:** satellite monitoring, deep learning, sea–land segmentation, shoreline detection, AI-based measurements, automatic labeled dataset construction, Sentinel-2, benchmark datasets

## Abstract

Monitoring the shoreline over time is essential to quickly identify and mitigate environmental issues such as coastal erosion. Monitoring using satellite images has two great advantages, i.e., global coverage and frequent measurement updates; but adequate methods are needed to extract shoreline information from such images. To this purpose, there are valuable non-supervised methods, but more recent research has concentrated on deep learning because of its greater potential in terms of generality, flexibility, and measurement accuracy, which, in contrast, derive from the information contained in large datasets of labeled samples. The first problem to solve, therefore, lies in obtaining large datasets suitable for this specific measurement problem, and this is a difficult task, typically requiring human analysis of a large number of images. In this article, we propose a technique to automatically create a dataset of labeled satellite images suitable for training machine learning models for shoreline detection. The method is based on the integration of data from satellite photos and data from certified, publicly accessible shoreline data. It involves several automatic processing steps, aimed at building the best possible dataset, with images including both sea and land regions, and correct labeling also in the presence of complicated water edges (which can be open or closed curves). The use of independently certified measurements for labeling the satellite images avoids the great work required to manually annotate them by visual inspection, as is done in other works in the literature. This is especially true when convoluted shorelines are considered. In addition, possible errors due to the subjective interpretation of satellite images are also eliminated. The method is developed and used specifically to build a new dataset of Sentinel-2 images, denoted SNOWED; but is applicable to different satellite images with trivial modifications. The accuracy of labels in SNOWED is directly determined by the uncertainty of the shoreline data used, which leads to sub-pixel errors in most cases. Furthermore, the quality of the SNOWED dataset is assessed through the visual comparison of a random sample of images and their corresponding labels, and its functionality is shown by training a neural model for sea–land segmentation.

## 1. Introduction

Coastlines are crucial ecosystems with both environmental and economic significance, as nearly half of the world’s population lives within 100 km of the sea [1]. These areas face various threats, including, fishing, pollution, shipping, and various consequences of climate change [2,3,4], making it imperative to monitor them for early detection of potential issues such as coastal erosion, that can cause harm to the environment and human settlements. Coastal monitoring can include detecting microplastics [5,6], and monitoring seagrasses [7,8], water quality [9,10], and antibiotics pollution [11,12] among others. Monitoring using in situ measurements is the most precise, but it can be costly and time-consuming, especially for large areas and/or frequent measurements. Remote sensing is an alternative solution that has evolved from aerial imagery taken from aircraft for the use of Unmanned Aerial Vehicles (UAVs) and Unmanned Underwater Vehicles (UUVs). Such methods of remote sensing offer advantages over in situ measurements, but still require extensive human intervention and specialized technologies.

More recently, satellite imagery has become a promising additional monitoring technique. Satellite data are characterized by global coverage and high temporal resolution and are often publicly accessible. Sentinel-2 and Landsat 8 are two of the most used Earth observation satellites, capturing multispectral images of the Earth’s surface with a resolution of up to 10 m. They provide, for a wide range of users including governments, academic institutions, and private companies, valuable information for monitoring changes in land cover and land use, as well as for detecting and mapping natural hazards [13,14,15]. The revisiting time of a few days enables near real-time monitoring of dynamic events on the earth’s surface. The increasing demand for high-quality earth observation data makes Sentinel-2 and Landsat 8 expected to remain key players in the earth observation satellite market in the future.

Lines delimiting water regions may be extracted from satellite images using traditional signal processing methods. Even in the AI era, these methods are valuable and often optimal tools to extract information from signals and images [16,17,18,19,20,21]. As regards the specific topic of coastline monitoring, edge detection algorithms are used in [16] for Sentinel Synthetic Aperture Radar (SAR) images, obtaining an extracted coastline with a mean distance of 1 pixel from the reference shoreline, measured through in situ analysis. In [22] coastline is achieved from very-high-resolution Pléiades imagery using the Normalized Difference Water Index (NDWI), which is one of the most popular techniques for automatic coastline extraction. NDWI is also used in [23], but, in this case, results are improved by using repeated measurements and adaptive thresholding. Another example of traditional signal processing for coastline detection is [18], where shorelines were extracted from multispectral images using a new water-land index that enhances the contrast between water and land pixels. Yet another example is [19], where unsupervised pixel classification is used for extracting shorelines from high-resolution satellite images.

Sentinel-2 satellite images are frequently employed for the purpose of coastline extraction, owing to their high spatial resolution and multispectral capabilities. In [24], shoreline changes in the Al Batinah region of Oman and the impact of Cyclone Kyarr are analyzed using Sentinel-2 images and the Digital Shoreline Analysis System (DSAS). In [25], the effectiveness of MODIS, Landsat 8, and Sentinel-2 in measuring regional shoreline changes is compared. Shorelines are extracted, again, with the DSAS and Sentinel-2 is identified as the most effective source of satellite images due to its higher spatial resolution. Another tool proposed for shoreline extraction is the SHOREX system [26,27]. It is able to automatically define the instantaneous shoreline position at a sub-pixel level from Landsat 8 and Sentinel 2 images. In [28], shoreline changes associated with volcanic activity in Anak Krakatau, Indonesia, are analyzed using a NDWI-based method on Sentinel-2 multispectral imagery.

In more recent years, semantic interpretation of images is being performed more and more by means of supervised machine learning, i.e., deep neural networks (DNN), due to its successful applicability in very different fields and to very different kinds of images, and shoreline extraction from satellite imagery is no exception [29,30]. The well-known U-Net architecture [31], in particular, is often used for effective DNN-based coast monitoring on a global scale [32,33,34,35,36,37,38,39,40]. Different types of satellite images have been used for this purpose, including Sentinel-1 SAR images [32,33,34], Landsat 8 and Gaofen-1 multispectral images [35,40], and true color images (TCI) from Google Earth [36,37,38,39]. In [41], eight deep learning models, including different variations of U-Net, are used for coastline detection, and their performances are compared. Other kinds of deep neural networks have also been proposed for coastline detection. In [42], ALOS-2 SAR images are analyzed using a densely connected neural network with two hidden layers. A multi-task network which includes both a sea–land segmentation and a sea–land boundary detection module, named BS-Net is instead proposed in [43].

The key requirement to successful supervised machine learning is, of course, the availability of large datasets of accurately labeled samples. The problem with coastline detection from satellite images is that datasets of appropriate size are not common. A possible solution is to build synthetic datasets, i.e., a collection of artificially generated realistic images, produced by a computer program together with the associated “exact” labels. Synthetic datasets have been built and used successfully in many applications [44,45,46], but their construction is unpractical for satellite images, which contain complex patterns difficult to reproduce realistically with computer graphics. This is true for TCI images and even more for images in other spectral bands. Manual labeling, based on visual interpretation of TCIs, is a long and boring task, but usable effectively as long as the shorelines are comparatively simple, e.g., with sea and land separated by a single line; when many images in the dataset have elaborate shorelines (as in the example that will be shown later), it becomes impractical and very burdensome. 

The present paper, extending the preliminary research in [47] (where a much smaller dataset is obtained), presents a method for automatically building a dataset of labeled satellite images for sea–land segmentation and shoreline detection. The method is based on the usage of publicly available shoreline data, together with publicly available satellite images. In particular, the method is developed to use shoreline data from the National Oceanic and Atmospheric Administration (NOAA) and satellite images from Copernicus Sentinel 2 project, obtaining the “Sentinel2-NOAA Water Edges Dataset” (SNOWED) [48], whose main features are the following.

- SNOWED is constructed with a fully automatic algorithm, without human intervention or interpretations.
- SNOWED is annotated using certified shoreline measurements.
- SNOWED contains satellite images of different types of coasts, located in a wide geographical area, including images related to very elaborate shorelines.

One intrinsic drawback of the automatic generation process is that some satellite images can contain water regions not included in the shoreline data used for labeling it and, hence, may have an incomplete label. This problem is however identified and handled as described in Section 4.1. 

With respect to other datasets of this type that have been proposed in recent years, the method presented in the paper to generate the SNOWED dataset is characterized by some innovative aspects. Datasets found in the literature are all based on the visual interpretation of satellite images and require therefore a strong human effort for labeling. Furthermore, the accuracy of sea/land segmentation labels depends directly on the quality of satellite images selected for the dataset and on how well they can be visually interpreted by humans. The methodology designed and implemented for this work uses instead independent measurements to automatically generate the labeled dataset, without any human intervention. This translates both to avoidance of tiresome human work and to the generation of sea/land labels having known and very low uncertainty. In addition, by using the proposed method, the source of satellite images (which is the Sentinel 2 project in the case of SNOWED) can be easily changed while using still the same shoreline measurements, leading to broader possibilities of application.

The paper is organized as follows. In Section 2, a review of the available datasets of satellite images for sea/land segmentation tasks is presented. In Section 3, the automatic dataset generation procedure is presented. In Section 4, results of the application of the proposed generation method are reported, together with quality assessment results. In Section 5 are the conclusions.

## 2. Publicly Available Datasets of Satellite Images for Sea/Land Segmentation

The aim of this section is to illustrate the already available public datasets developed for training deep learning models for sea/land segmentation. Particular attention is dedicated to the general characteristics of the provided datasets and to the generation process used to obtain them. This is useful to understand the novelty and relevance of the proposed work, and to conveniently compare the proposed SNOWED dataset with the other available alternatives. 

Two major features are considered for each dataset: the number of samples containing both water and land pixels, and the source of labeling information. These features are indeed the only ones strictly related to the effectiveness of a dataset for training a neural network. We consider the number of images containing both land and sea, rather than the absolute number of images, because samples containing only one class can be trivially extracted from large areas that are known to contain only sea or only land. Besides, we highlight that the source of labeling information determines the accuracy of the labels, and hence the accuracy of models trained using the dataset.

### 2.1. Water Segmentation Data Set (QueryPlanet Project)

The water segmentation data set [49] has been created as a part of the QueryPlanet project, which has been funded by the European Space Agency (ESA). The dataset is composed of satellite images of size 64×64 from the Sentinel-2 Level-1C product. Each of them has been manually labeled by volunteer users of a collaborative web app. Volunteers were prompted with an initial label obtained by calculating the NDWI [50] and had to visually compare it with the corresponding satellite TCI and correct eventual discrepancies based on their interpretation of the image. The online labeling campaign led to the creation of 7671 samples, but only 5177 of them contain both sea and land pixels.

### 2.2. Sea–Land Segmentation Benchmark Dataset

The dataset proposed in [51] contains labeled Landsat-8 Operational Land Imager (OLI) satellite images, of different types of Chinese shorelines: sandy, muddy, artificial and rocky coasts. The labels of the dataset are obtained through a multi-step human annotation procedure. First, Landset-8 OLI images with less than 5% cloud cover are selected along the Chinese shoreline. These images are pre-processed by applying radiometric calibration and atmospheric correction and then are manually annotated by dividing all their pixels into two classes: sea and land. Finally, satellite images are cut into small patches and each patch is checked to remove the defective ones (e.g., blank and cloud-covered patches). At the end of the procedure, 3361 images of size 512×512 are obtained, but only 831 of them contain both classes. 

### 2.3. YTU-WaterNet

The YTU-WaterNet dataset proposed in [52] contains Landsat-8 OLI images too. The dataset is created starting from 63 Landsat-8 OLI full-frames containing coastal regions of Europe, South and North America, and Africa. Only the blue, red and near-infrared bands are used for the samples of the dataset to reduce the dataset size and the computational load needed for training operations. The satellite images are cut into 512×512 patches and binary segmented by exploiting OpenStreetMap (OSM) water polygons data [43]. OSM data is created by volunteers based on their geographical knowledge of the area or on visual interpretation of satellite images. This data is available as vector polygons, which are then converted to raster images representing the water regions of the sample. Finally, a filtering operation is performed to eliminate cloud-covered samples and samples with only one class, while samples with a mismatching label are identified and eliminated by visual inspection. The YTU-WaterNet dataset contains 1008 images.

### 2.4. Sentinel-2 Water Edges Dataset (SWED)

The most recent dataset is the Sentinel-2 Water Edges Dataset (SWED), proposed in a research work supported by the UK Hydrographic Office [53]. SWED uses Sentinel-2 Level-2A imagery, semantically annotated through a semi-automatic procedure. The first step of the dataset creation process is the selection of Sentinel-2 images between 2017 and 2021. Only clear and cloud-free images are selected, by filtering on the ‘cloudy pixel percentage’ metadata associated with each image, and then by visually inspecting the obtained search results. Furthermore, images are manually selected to cover a wide variety of geographical areas and types of coasts. A water/non-water segmentation mask is therefore created for each of the selected Sentinel-2 images. First, a false color image with visually good contrast between water and non-water pixels is searched by trial and error among those that can be obtained by rendering different combinations of Sentinel-2 bands in the RGB channels. The selected combination of bands is not the same for each image, although three combinations are found to be a good starting point. Secondly, a manually refined k-means-based procedure is applied to the rendered false color images to collect their pixels into two clusters, corresponding to water and non-water regions. Finally, segmentation masks are manually corrected by visual comparison against high-resolution aerial imagery available on Google Earth and Bing Maps. This imagery is, however, obtained as a composition of multiple images acquired on different and not precisely known dates, and therefore in some cases can represent inaccurately the actual state of the coasts at the Sentinel-2 acquisition time. The SWED dataset contains 26,468 images of size 256×256, cut from the annotated Sentinel-2 full-tiles, but only 9013 of them contain both classes.

### 2.5. Summary of the Characteristics of the Already Available Datasets, and of the New SNOWED Dataset

The described datasets are the result of a very intense effort and provide solid solutions for training and benchmarking machine learning models for shoreline recognition. Of course, a larger number of samples, or another dataset that can be used together with them, is desirable. Another improvable feature is the labeling process accuracy: a specific quality assurance on the shoreline labels would be a clear plus. 

The methodology described in this work aims precisely at these goals: providing further samples useful for training neural models for satellite coastline measurements, along with labels coming from certified coastline measurements.

Table 1 summarizes the main characteristics of the four datasets in the literature described in this section, and those of the SNOWED dataset obtained with the procedure illustrated in the present work. The number of images reported in Table 1 refers to images containing both sea and land classes. As can be seen, the image size is not the same for all the datasets and therefore a conversion is needed to directly compare the number of images of the two datasets. For example, the 1008 512×512 images of YTU-WaterNet correspond to 4032 256 × 256 images.

## 3. Data and Methods

The methodology presented in this paper consists in combining publicly available satellite images and shoreline data. This is a non-trivial task since many preprocessing operations and quality checks are needed to obtain accurately annotated samples. The methodology, and the involved processing, are illustrated with the concrete construction of a dataset, where the source of satellite imagery is the Level-1C data product of the Sentinel-2 mission, and the source of shoreline data is the Continually Updated Shoreline Product (CUSP).

### 3.1. Data Sources

#### 3.1.1. Sentinel-2 Satellite Imagery

The Sentinel-2 mission consists of a constellation of two satellites for Earth observation, phased at 180° to each other to provide a revisit period of at most 5 days. The satellites are equipped with the MultiSpectral Instrument (MSI) that acquires images in 13 spectral bands, with spatial resolutions up to 10 m (four bands have a resolution of 10 m, six bands have a resolution of 20 m, three bands have a resolution of 60 m). Level-1C data products provide Top-Of-Atmosphere reflectances measured through the MSI as 100×100 km^2^ ortho-images (tiles) in UTM/WGS84 projection [54]. Level-1C data covers all continental land and sea water up to 20 km from the coast, from June 2015 to the current date [55].

Sentinel-2 data has been selected since this mission provides better performances compared to other public continuous Earth observation missions, in terms of both spatial resolution and revisit period. Landsat 8/9 mission, for example, has a spatial resolution of at most 15 m and a revisit period of 8 days. The choice of satellite imagery data sources, however, is not a conditioning factor for the dataset creation process and other products can be used with few trivial changes in the procedure.

#### 3.1.2. Shoreline Data

CUSP is developed by U.S. NOAA with the aim of providing essential information to manage coastal areas and conduct environmental analyses. This dataset includes all continental U.S. shoreline with portions of Alaska, Hawaii, the U.S. Virgin Islands, Pacific Islands, and Puerto Rico. CUSP provides the mean-high water shoreline, measured through vertical modeling or image interpretation using both water level stations and/or shoreline indicators. All data included in CUSP is verified by contemporary imagery or shoreline from other sources [56]. Another important feature of CUSP is that the shoreline is split into shorter paths and each of them has additional information associated, including the date and type of source data used to measure the shoreline, the type of coast and the horizontal accuracy, which represents the circular error at the 95% confidence level [57]. An analysis of the horizontal accuracy shows that 90% of paths have measurement errors ≤ 10 m, while 99.97% of paths have measurement errors ≤ 20 m. NOAA’s CUSP paths have therefore a very high accuracy, comparable and, in most cases, overcoming the resolution of Sentinel 2 imagery. To our knowledge, NOAA’s CUSP is the only publicly available source of shoreline data with these features, which are essential to perform the dataset generation procedure proposed in this work. In principle, nothing prevents one from using other sets of shoreline measurements, with the same essential features, i.e., geographic coordinates, date, high accuracy, and possibly the measurement method.

### 3.2. Shoreline Data Preprocessing (Selection and Merging)

**Figure 1:**
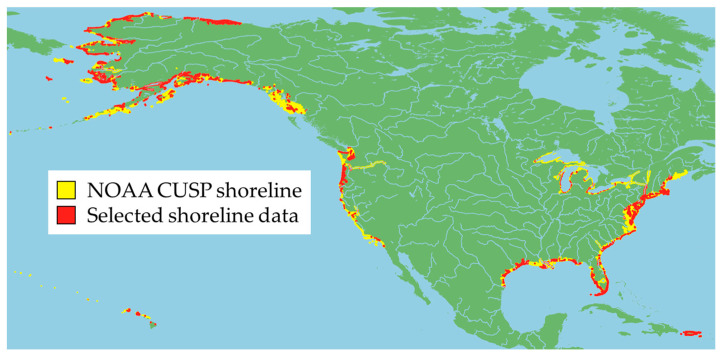
Selected CUSP shorelines (those from June 2015 onwards) compared to the complete data.

A preliminary filtering operation is performed on CUSP data to exclude shoreline that has been extracted from observations prior to the Sentinel-2 mission launch in June 2015. A representation of the shoreline remaining after this preliminary operation is depicted in the map of North America in Figure 1 which shows that locations of useful shorelines are very heterogeneous, spanning most areas of the U.S. coast. This is an advantageous feature since it guarantees a great variability of the satellite images included in the final produced dataset.

Selected shoreline paths that share one terminal point and have the same date are then merged, in order to optimize the satellite images selection procedure described in the following. Statistics about the NOAA CUSP shoreline data and the selected paths are reported in Table 2.

### 3.3. Selection of Satellite Images

In the practical implementation of the procedure, we have obtained the Sentinel-2 Level-1C tiles of our interest, which are 10,980×10,980 pixels, using the *Plateforme d’Exploitation des Produits Sentinel* of the *Centre National d’études Spatiales* (PEPS CNES) [58]. 

Satellite images are selected on the basis of the location and the date of the merged shoreline paths obtained in the former step. It is important here to clarify the issue of *dates and times* of satellite images and on-field measurements used for labeling.

Obviously, the ideal situation is to have perfect simultaneity between the acquisition of the satellite image and the field measurements of the area it takes. It is easy to understand, however, that this situation is unfeasible and never occurs in practice. 

Even when in situ measurements are made specifically for image labeling, simultaneity is not achieved in practice (see e.g., [7]). Satellite images, indeed, are taken at intervals of some days (5 days in the case of Sentinel-2), and an image is not always usable, due to the presence of clouds or other causes: hence, usable images have dates that cannot be chosen as desired by the user and can be spaced between them by many days. For this same reason, satellite monitoring is not designed to keep track of changes that occur in a few hours, but of changes over months and years. In general, one must always choose the image *with the date nearer to that of interest*. When labeling a dataset, the date of the image must be as close as possible to that of the in situ measurement.

On the basis of the above consideration, PEPS CNES has been queried according to the following criteria.

- Sentinel tiles must contain the shoreline path.
- Cloud cover of Sentinel tiles must be lesser than 10% (parameter: *cloud_cover*).
- Sentinel tiles acquisition date must be at most 30 days (parameter: *time_difference*) before or after the shoreline date.

The time difference between satellite images and NOAA CUSP measurements is exactly known and recorded in the dataset. It never exceeds the parameter *time_difference*: otherwise, the data sample is not generated. When more than one result is obtained, the tile having the acquisition date closest to the shoreline date is selected.

It is possible to choose different values for the parameters *cloud_cover* and *time_difference*. A value of *cloud_cover* > 10% generates more dataset samples, but it is more likely that they will be discarded in the following steps (see Section 3.4), due to the presence of clouds. A parameter *time_difference* < 30 days generates fewer dataset samples, but the shorelines in the obtained images are likely to be negligibly different since the resolution of Sentinel-2 is 10 m / pixel, and therefore only changes of the order of tens of meters are important. In any case, a *time_difference* < 10 days is not reasonable due to the revisit time of Sentinel-2.

The selection obtained with these constraints has been found to provide a good compromise between the computational resources required to generate the dataset (directly related to the number of selected tiles) and the final size of the dataset, which can grow if more tiles, and hence more shoreline paths, are considered. It is worth highlighting that:

(1) The quality of each sample generated with this method is assured by later checks, which are also automatic, being based on Sentinel data themselves (see Section 3, in particular, Section 3.4). For example, the presence of clouds in localized areas of the tile is not detrimental.
(2) Further releasing the constraints (cloud coverage and time difference) do not lead, ultimately, to a significant increase in the dataset’s size.

The described procedure has obtained 987 tiles, containing 102,283 shoreline paths (about 20% less than the overall number of paths).

### 3.4. Extraction of Samples and Labeling

The selected Sentinel-2 tiles are then downloaded and processed singularly to extract the semantically annotated samples. Two preliminary operations are performed before the actual extraction phase. 

First, Level-2A products are generated from Level-1C products using the sen2cor processor [59]. Level-2A products are composed of (i) a scene classification (SC) mask and (ii) surface reflectance obtained through atmospheric correction [60]. The SC mask assigns one of 12 classes (including water, vegetated and non-vegetated land, and clouds) to each pixel of the tile and is the only data needed in subsequent steps. 

Second, the shoreline paths associated with each Sentinel tile are projected to the plane of the UTM/WGS84 zone containing the Sentinel tile. The UTM coordinates of the vertices of the Sentinel tile in the plane of the UTM zone are also known (they can be downloaded from [61]), and thus the pixel coordinates of the shoreline points inside the tile can be computed.

**Figure 2:**
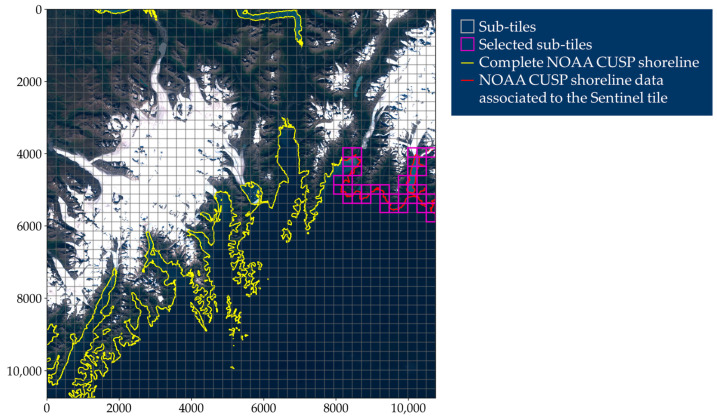
Example of Sentinel-2 Level-1C tile (true color image) split into sub-tiles of size 256×256. Only sub-tiles containing shoreline based on measurements performed in dates within 30 days from the Sentinel tile’s acquisition date are analyzed.

The 10,980×10,980 pixels Sentinel tile is then split into sub-tiles of size 256×256, among which the samples of the dataset are selected. The sub-tile size has been chosen so that a direct comparison, and a side-to-side utilization, is possible of the obtained dataset and that described in [53], the most recent and numerous for water segmentation in the literature. In Figure 2 an example of a sub-tiling grid is depicted.

The subsequent processing involves only sub-tiles containing shoreline paths, as shown in Figure 2. The basic task is to create, for each sub-tile, a binary segmentation map based on NOAA CUSP shoreline paths. This operation is of some complexity and must be illustrated in detail.

First of all, we specify that, for any sub-tile, all shoreline paths partially or completely contained in it are considered, *independent of their date*. A strict constraint on the dates of all the used shoreline segments leads to discarding many sub-tiles, due to the short shoreline segments with dates too different from that of the tile. Instead, completing the shoreline including also short segments measured in different dates allows the construction of a dataset with much more sample, without compromising meaningfully the quality of the shoreline data. Besides, the date of each shoreline path is supplied in the dataset, so that samples can be later selected, if deemed useful, according to arbitrary constraints on the time difference between the Sentinel date and the CUSP shoreline dates.

**Figure 3:**
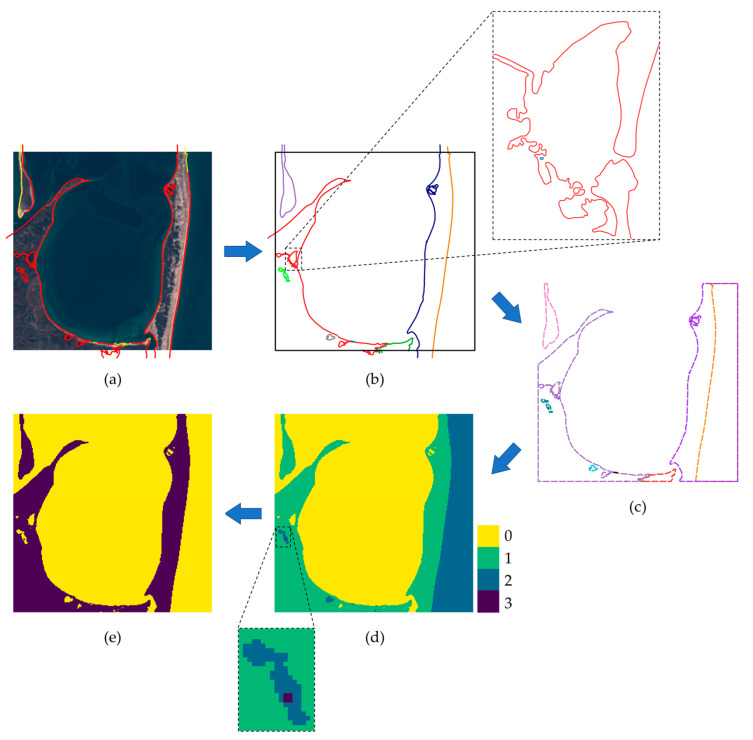
Steps for generating the binary segmentation mask for a sample of the dataset. (**a**) Selection of the shoreline paths inside the sub-tile. Paths with dates compatible with the Sentinel-2 tile’s date are depicted in red and the other paths in yellow. (**b**) Merging of contacting paths. Distinct merged paths are depicted using different colors, while the sub-tile border is in black. (**c**) Clipping of merged paths using the sub-tile border as the clipping window. After this stage, closed polygons are obtained. (**d**) Starting from a zero-filled matrix of the sub-tile, ones are added in the regions defined by the polygons. (**e**) A binary map is obtained by classifying the pixels of matrix (**d**) into even and odds.

The process used for generating the binary segmentation mask of sub-tiles from CUSP shoreline paths is depicted in Figure 3. As a first step, shoreline paths completely or partially contained in the sub-tile are selected (Figure 3a). The second step is to merge contacting paths: merged paths, depicted with different colors in Figure 3b, can be closed (e.g., the light green path and the gray path) or open (e.g., the orange, blue and red paths). If open paths have an end inside the sub-tile, the sub-tile is discarded; otherwise, paths are clipped using the sub-tile borders as the clipping window. Only closed polygons are obtained after this operation, as shown in Figure 3c. To obtain a binary mask, polygons are filled with ones and summed, producing the matrix in Figure 3d; finally, a binary map (Figure 3e) is obtained by selecting the even and odds elements of the matrix in Figure 3d.

**Figure 4:**
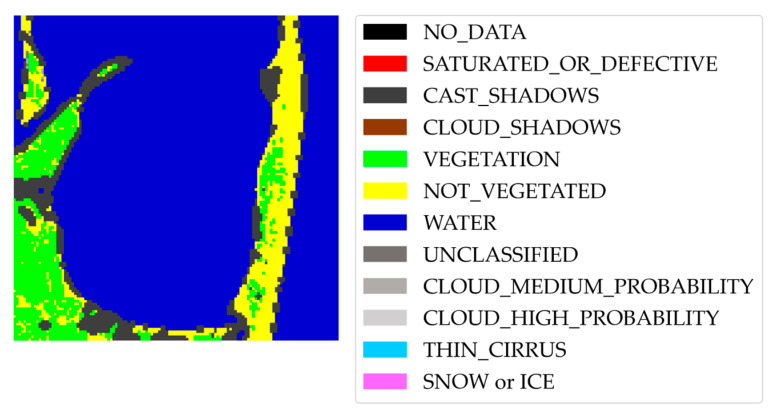
Sentinel-2 Level-2A scene classification (SC) mask of the sub-tile in Figure 3. For the sake of clarity, the legend includes all the 12 classes provided by Level-2A SC, although only some of them are identified in this case.

The segmentation label of the sub-tile is created by assigning water and land categories to the two classes of the mask in Figure 3e based on the previously computed Level-2A SC layer, depicted in Figure 4 for the case considered in Figure 3. The class in the mask containing more Level-2A water pixels is categorized as water, while the class containing more non-water Level-2A pixels is categorized as land. The SC layer is also used to evaluate the correctness of the label. In particular, the sub-tile is discarded if the water class and land class contain less than 80% Level-2A water pixels and non-water pixels, respectively.

### 3.5. Overview of the Dataset Generation Procedure

**Figure 5:**
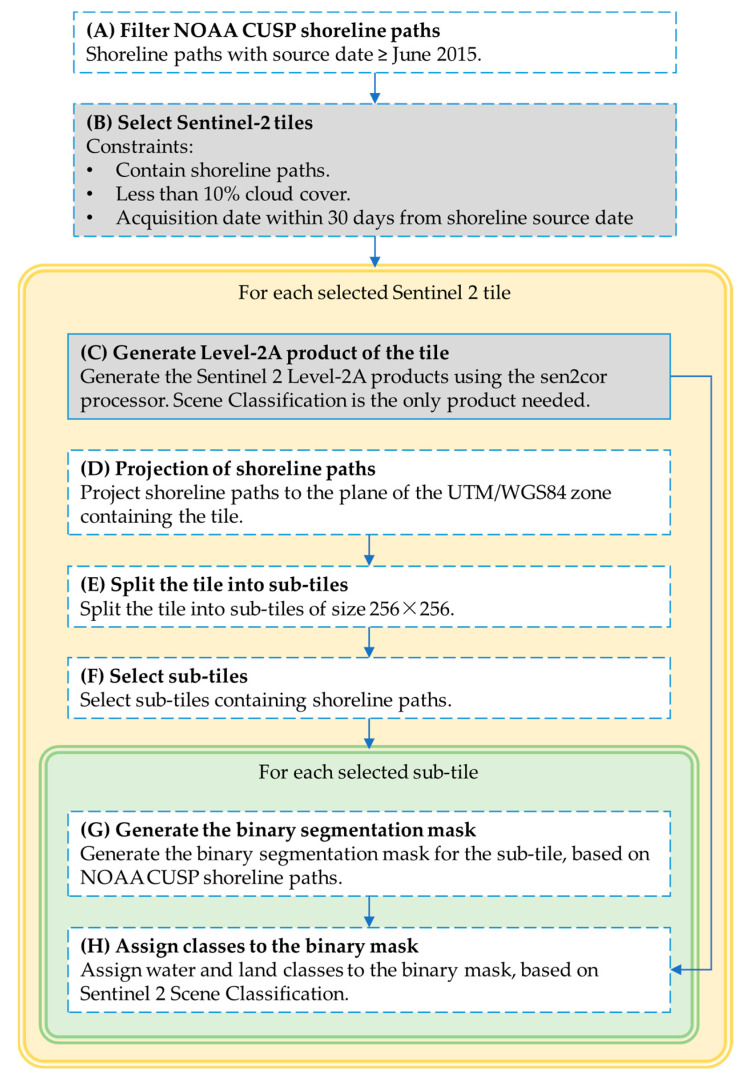
Flowchart of the proposed method. Details about each operation are provided in the previous subsections. Operations performed with novel methods, proposed in the paper, are marked with a dashed blue line. Operations performed with known methods are marked with a solid blue line. Operations with gray backgrounds are those specific for Sentinel 2 imagery.

For the sake of clarity, a flowchart of the previously described dataset generation method is depicted in Figure 5. The proposed method is an original solution, and each step except one (marked with a solid line) has been designed and implemented purposely for this work. The flowchart highlights the novelty of this method compared to others reported in the literature and reviewed in Section 2. In these works, sea/land labeling is fundamentally based on the human interpretation of satellite imagery, while the proposed method uses shoreline measurements from NOAA CUSP as a source for automatic labeling.

In the flowchart in Figure 5, operations with a gray background, namely B and C, are specific to Sentinel 2 and need major refactoring if other imagery sources are used. All the other operations require instead only trivial changes. The general logic and overall procedure for dataset generation are, however, the same even if other imagery sources are used. Furthermore, the changes required for operations B and C are not substantial. In particular, for operation B, the same constraints for selecting satellite images must be used to query the appropriate satellite imagery platform (PEPS CNES is used in this work for Sentinel images). For operation C, the only required output is a low-accuracy sea/land classification of the satellite image, used later in operation H, and this can be easily obtained e.g., by computing the NDWI.

## 4. Results and Discussion

The annotated dataset generated using the proposed method counts 4334 samples of size 256×256 containing both water and land pixels. The dataset has been built retrieving all 13 Sentinel-2 MSI bands, which are therefore all present in each sample. The resolution of Sentinel-2 images is different for the various bands, the best being 10 m per pixel. The images in all 13 bands have been linearly rescaled to a uniform 10 m per pixel spatial resolution, a standard operation that allows one to have all the images in a single 3-d array. Each sample is provided with the water/land segmentation label and with the following additional information.

- Level-2A SC mask.
- Shoreline paths are used for labeling, each with its measurement date.
- PEPS CNES identifier of the Sentinel-2 Level-1C tile.
- Acquisition date of the Sentinel-2 Level-1C tile.
- Pixels offset of the sub-tile in the complete Sentinel-2 image.

**Figure 6:**
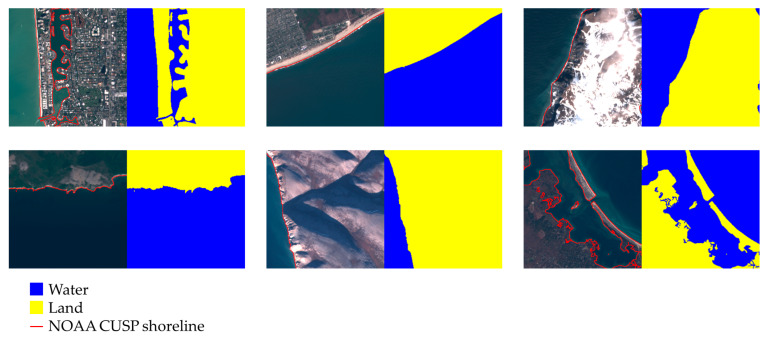
Examples of semantically annotated Sentinel-2 satellite images (true color image on the left) and labels (on the right) contained in the proposed dataset.

Some examples taken from the generated dataset are depicted in Figure 6. It is possible to appreciate the accuracy of the labeling, especially in the two cases of complicated water edges.

In the next subsections, the quality of the dataset is assessed both visually, by comparing images of a random subset of the dataset with their corresponding labels, and from a functional point of view, by training and testing a deep learning model using SNOWED.

### 4.1. Dataset Visual Quality Assessment

In premise, it is important to remember that *any* dataset is prone to including inconsistent labels. In datasets that are manually labeled by subject matter experts there is room for human mistakes and subjective interpretations; in automatically labeled datasets problems may arise from intrinsic imperfections in the labeling algorithm. The problem of inconsistent labels is negligible only in synthetically generated datasets, which, in contrast, are prone to providing samples not similar enough to the actual samples with which the model must work. Therefore, assessing the quality of a dataset, and providing methods to improve it, can be considered a good metrological practice, always advisable.

The automatic method presented here to construct the dataset, together with its clear advantages, has an intrinsic drawback, that occasionally produces samples with incomplete labels. The problem arises from the fact that, in general, there is no guarantee that *the set of measured shorelines includes all the water edges in each sample*. In the case of SNOWED, a Sentinel-2 sub-tile may include water edges that have not been measured and included in CUSP NOAA. This problem can be avoided only by using a (hypothetical) collection of shorelines that is guaranteed to include *all* the water edges present in a large enough geographic region. This is not the case with NOAA CUSP.

Because of the considerations above, we provide here a procedure to check, assess and improve the dataset quality.

**Figure 7:**
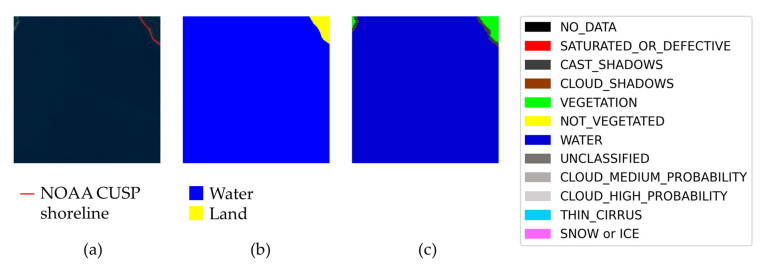
Examples of “bad” samples with a clearly incomplete label, found in the process of quality assessment of the dataset. (**a**) True color image. (**b**) Sea/land segmentation. (**c**) Sentinel-2 Level-2A scene classification. There is a portion of land not included in the label, in the upper left corner.

Any single sample of the dataset can be checked by inspecting three images provided in it, i.e., the TCI, the label, and the Sentinel-2 scene classification, as shown in Figure 7. The TCI and the label are visually compared, and the Sentinel-2 SC is used as a guide. It is important to remember that the latter image may only serve as a guide for a human, and not for an automatic check: the scene classification is, indeed, not very accurate, and in some cases misclassifies regions of the satellite image. 

In Figure 7 it is clear that, in the upper left corner, there is a small water edge, and therefore a small portion of land, not included in the label. This water edge was simply not present in NOAA CUSP and is of a length comparable with that of the labeled water edge in the sample. This sample, therefore, is considered “bad”. 

**Figure 8:**
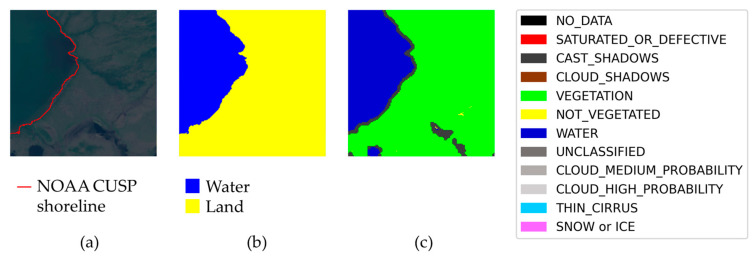
Example of a “suspect” sample, with a possibly incomplete label. There is a region that is water according to Sentinel-2 scene interpretation. The main shoreline in the sample is labeled. (**a**) True color image. (**b**) Sea/land segmentation. (**c**) Sentinel-2 Level-2A scene classification.

In Figure 8, instead, there is a sample that we consider “suspect”. In this sample, the main shoreline is clear and labeled, but there is a small region which is classified as water by Sentinel-2 SC and as land according NOAA CUSP. It is not obvious if the water edge is real or not—a further check with an independent source should be made, e.g., using commercial satellite imagery with very high resolution—and the (possibly) missing edge is much shorter than the labeled one.

**Figure 9:**
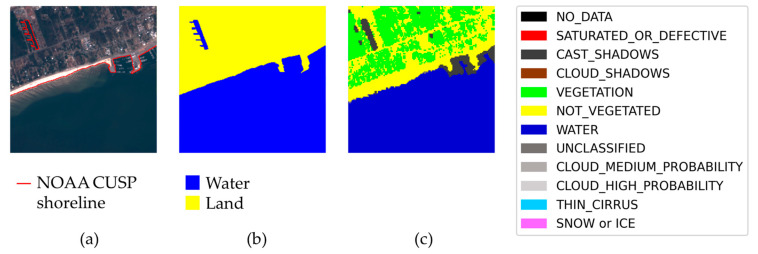
Example of a “particularly good” sample, with an elaborated water edge that is ignored by Sentinel-2 SC. (**a**) True color image. (**b**) Sea/land segmentation. (**c**) Sentinel-2 Level-2A scene classification.

We want to highlight that, together with “bad” and “suspect” samples, the dataset has many samples that are “particularly good”, in the sense that the label includes elaborated shorelines, difficult to recognize by a human, and that the label is also completely ignored by Sentinel-2 SC. An example is in Figure 9.

We have assessed the dataset quality by checking a randomly selected subset of n=200 samples, out of N=4334 sample total. In the selected samples, we found five “bad” samples, with clearly incomplete labels, and 30 “suspect” samples, with possibly incomplete labels and ambiguous interpretation. The point estimate of the fraction of “bad” and “suspect” samples in a dataset (a conservative estimate of the fraction of improvable samples) is therefore:

$$\hat{p}=\frac{35}{200}=17.5\%$$

and the 95% confidence interval for this fraction is, approximately:

$$p_{95\%}=\hat{p}\pm 1.96\sqrt{\frac{\hat{p}\left(1-\hat{p}\right)}{n}\frac{N-n}{N-1}}=\left(17.5\pm 5.1\right)\%$$

where the Gaussian approximation of the hypergeometric distribution has been applied, taking into account the term (N−n)/(N−1) to correctly account for the sampling without replacement in the acceptance sampling procedure [62,63].

The samples in this fraction can be further processed, by humans or by an algorithm using a different source of water edge data, to improve the labeling. It can also be discarded, even if this choice does not seem appropriate for the “suspect” samples, whose labels always include the “main” shorelines in the image.

In the next subsection, the dataset is used “as is”, without discarding or correcting neither the bad samples nor the suspect ones found in the assessment process. It is shown that the dataset trains quite effectively a simple neural model for shoreline detection. 

### 4.2. Example Application of the Dataset

The SNOWED dataset has been employed to train a standard U-Net segmentation model [31]. The dataset has been shuffled and then split into a training and a validation subset, corresponding to 80% and 20% of the samples respectively. Afterwards, the U-Net neural network has been trained for 30 epochs, using the Adam optimizer [64]. Cross-entropy has been used as a loss function in the optimization, while mean intersection over union (IoU) is the metric to evaluate the performance of the neural model.

**Figure 10:**
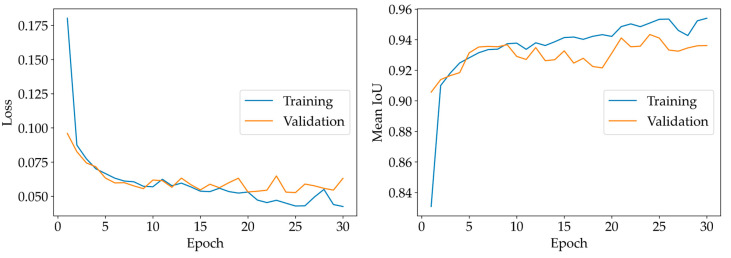
Loss function and mean IoU versus number of training epochs.

**Figure 11:**
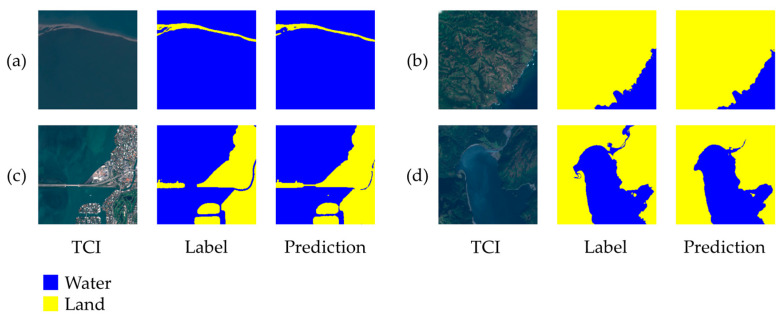
(**a**–**d**) Sea/land segmentation results obtained with the trained U-Net model for the first four samples of the validation set.

The training process is depicted in Figure 10. The final mean IoU for the validation set, obtained after completing the training phase, is 0.936. In Figure 11, the sea/land segmentation masks produced by the trained U-Net model for the first four samples of the validation set are depicted. The visual inspection of these results shows that an accurate sea/water segmentation is obtained, even if we used the standard U-Net model without any further optimization.

## 5. Conclusions

Measuring boundaries between land and water is important for understanding and managing environmental phenomena like erosion, accretion, sea level rise, etc. Measurements from satellite imagery are particularly useful to provide consistent information over large areas and long periods of time (even if with limited spatial resolution). There is not a single best method to measure shorelines using satellite data, but artificial intelligence techniques are also acquiring more and more importance in this field. Therefore, recent research is devoted to the construction of datasets of satellite images with shoreline labels, customarily obtained with human work of image interpretation and annotation. Constructing datasets with human intervention has obvious costs and drawbacks, which are especially meaningful considering that a dataset of labeled images of a given satellite cannot be used to work with images of other satellites.

Based on these considerations, we have focused on the task of constructing a labeled dataset of satellite images for shoreline detection *without any human intervention*. The algorithm uses NOAA CUSP shoreline data to properly select and annotate satellite images. By annotating Sentinel-2 Level-1C images, the algorithm has constructed the SNOWED dataset, which can be used alongside the very recent SWED dataset. With minimal adjustments, the algorithm can be used to construct datasets for different satellites. 

The concept and results presented in this work show that it is possible, in general, to construct readily a dataset of labeled satellite images, if a set of in situ measurements with geographic and temporal data are available. Therefore, satellite monitoring and measurements can receive a great boost by increasing the public availability of measurement data coming from accurate ground surveys.

## Figures and Tables

**Table 1:** Main characteristics of the four examined datasets. The same characteristics for the new SNOWED dataset are included for comparison. They highlight that SNOWED (i) is compatible with, and adds up, to SWED; (ii) uses NOAA measurements, instead of human interpretation of images.

Dataset ID	N. of Images	Image Size	Source of Coastline Data
QueryPlanet [49]	5177	64 × 64	Human interpretation of TCI images
Sea–land segmentation benchmark dataset [51]	831	512 × 512	Human interpretation of TCI images
YTU-WaterNet [52]	1008	512 × 512	Human-generated OpenStreetMap water polygons data
SWED [53]	9013	256 × 256	Human interpretation of high-resolution aerial imagery available in Google Earth and Bing Maps
SNOWED	4334	256 × 256	U.S. NOAA shoreline measurements

**Table 2:** Statistics about the NOAA’s CUSP shoreline data.

Initial number of paths	779,954
Total length of the paths	403,707 km
Number of selected paths	221,331
Total length of selected paths	107,600 km
Number of paths after merging	126,938

## Data Availability

The data presented in this study are openly available in Zenodo at https://doi.org/10.5281/Zenodo.7871636, reference number [48].
